# Computational Tools for Investigating Pathogen, Pathogen-Host Interaction, and Infectious Disease

**DOI:** 10.1155/2018/5476875

**Published:** 2018-07-18

**Authors:** Jialiang Yang, Bo Liao, Jianqiang Ye, Taoyang Wu

**Affiliations:** ^1^College of Information Engineering, Changsha Medical University, Changsha, Hunan 410219, China; ^2^Department of Genetics and Genomic Sciences, Icahn School of Medicine at Mount Sinai, New York, NY 10029, USA; ^3^College of Information Science and Engineering, Hunan University, Changsha, Hunan 410082, China; ^4^Ministry of Education Key Lab for Avian Preventive Medicine, Yangzhou University, Yangzhou 225009, China; ^5^School of Computing Sciences, University of East Anglia, NR4 7TJ Norwich, UK

Recent developments of high-throughput sequencing technologies and systems biology methods open novel opportunities to investigate pathogen, pathogen-host interactions, and infectious diseases at system-wide scales. For example, the tool antigenic map has been adopted by Centers for Disease Control and Prevention in the US to help select vaccine strains for seasonal influenza each year. Disease transmission models have been used to study and help inform public health interventions for a few infectious diseases for human and animals. However, the clinical application of computational tools is still on its early stage. With more and more data available for pathogens and their hosts at genome, transcriptome, proteome, methylome, and epigenome levels, computational modelling, big data analysis, and systems biology have become more and more important in revealing the biological mechanisms underlying these data, unraveling the secrets behind pathogen evolution, infection, and pathogen-host interactions.

In this special issue, we have received 18 papers, out of which 7 has been accepted for publication.

Firstly in a review article, G. Mboowa et al. described a range of host-pathogen genomic loci that have been associated with common infectious disease (e.g., HIV/AIDS, tuberculosis, and malaria) susceptibility and resistant patterns in the era of high-throughput sequencing. They further highlighted potential opportunities for the applications of these genetic markers in therapeutic development involving genome-editing technologies like CRISPR/Cas9.

X.-m. Wu et al. investigated the genomic sequence and pathogenicity of a pseudorabies virus that was isolated during the 2011-2012 outbreak in Fujian Province, China. Through bioinformatics and pathogenic studies, the authors found that the FJ-2012 strain is highly pathogenic and identified genetic variations compared with the reference sequence. This study provided useful information to understand the evolution and pathogenicity of pseudorabies viruses.

L. Zheng et al. studied the interactions between p2 of rice stripe virus and three functional domains of NbFib2 including the N-terminal fragment containing a glycine- and arginine-rich (GAR) domain, the central RNA-binding domain, and C-terminal *α*-helical domain using yeast two-hybrid, colocalization, and bimolecular fluorescence complementation (BiFC) assays. Their results suggested that the N-terminal domain is indispensable for NbFib2 to target the nucleolus and cajal body, p2 binds to all three regions of NbFib2, and they target to the nucleus but fail to target the nucleoli and cajal bodies.

D. Xiao et al. first sequenced the whole genome of *Sparassis latifolia*, an important antidiabetic, antihypertension, antitumor, and antiallergen fungus. They then depicted critical enzymes involving in carbohydrate and glycoconjugate metabolism as well as secondary metabolite and performed the comparative and phylogenetic analyses of this fungus and annotated the predicted genes, which sheds some light on the genetic basis of the medical properties of *Sparassis latifolia*.

B. Gao et al. adopted combined methods of bioactive trace technology and phytochemical extraction and separation, to guide the isolation and purification of the effective chemical constituents on the water-soluble components of aerial parts of *Isatis tinctoria*, one of the most well-studied Chinese herbs effective against the dengue fever. They were capable of identifying and inferring the structures of 27 types of chemical compounds named GB-1, GB-2,…, and GB-27, respectively, among which GB-8 is a novel compound. Further study of these compounds is critical to reveal the secrets behind the medicinal effects of *Isatis tinctoria*.

Z. Chen et al. identified a series of microsatellite markers of *O. nipae*, a type of nipa palm hispid beetle originated in Malaysia through transcriptomic and polymorphic study, and tested the genetic differences of two Fujian *O. nipae* populations in China through nine newly developed microsatellite markers. They found that these polymorphism microsatellite markers are powerful tools for pests' population genetic and dispersal studies. It is the first time that microsatellite markers have been utilized in *O. nipae*.

Finally, A. Endo and H. Nishiura presented a multisite multispecies transmission model along East Asian-Australian Flyway to study the role of migration in maintaining the transmission of avian influenza in waterfowl. They found that mallard was the most important host with the highest transmission potential, and high- and middle-latitude regions appeared to act as hotspots of influenza transmission. In addition, their study suggested that the prevalence of avian influenza in Oceania region is dependent on the inflow of infected birds from other regions.

